# A IR-Femtosecond Laser Hybrid Sensor to Measure the Thermal Expansion and Thermo-Optical Coefficient of Silica-Based FBG at High Temperatures

**DOI:** 10.3390/s18020359

**Published:** 2018-01-26

**Authors:** Litong Li, Dajuan Lv, Minghong Yang, Liangming Xiong, Jie Luo

**Affiliations:** 1 State Key Laboratory of Optical Fiber and Cable Manufacture Technology, Yangtze Optical Fibre and Cable Joint Stock Limited Company, Wuhan 430073, China; lvdajuan@yofc.com (D.L.); xiongliangming@yofc.com (L.X.); luojie@yofc.com (J.L.); 2 National Engineering Laboratory for Fiber Optic Sensing Technology, Wuhan University of Technology, Wuhan 430070, China

**Keywords:** fiber optics sensor, IR-femtosecond laser, high temperatures, thermal expansion coefficient, thermo-optical coefficient

## Abstract

In this paper, a hybrid sensor was fabricated using a IR-femtosecond laser to measure the thermal expansion and thermo-optical coefficient of silica-based fiber Bragg gratings (FBGs). The hybrid sensor was composed of an inline fiber Fabry-Perot interferometer (FFPI) cavity and a type-II FBG. Experiment results showed that the type-II FBG had three high reflectivity resonances in the wavelength ranging from 1100 to 1600 nm, showing the peaks in 1.1, 1.3 and 1.5 μm, respectively. The thermal expansion and thermo-optical coefficient (1.3 μm, 1.5 μm) of silica-based FBG, under temperatures ranging from 30 to 1100 °C, had been simultaneously calculated by measuring the wavelength of the type-II FBG and FFPI cavity length.

## 1. Introduction

Fiber Bragg gratings (FBGs) are becoming increasingly important in high temperature application fields such as aerospace engineering, chemical aggressive and energy fields, due to their light weight, immunity to electromagnetic interference, durability against harsh environments, and fast response [1,2]. The induced local material changes in silica fiber yield an increase of the refractive index. FBGs are defined as type-I or type-II depending on whether the exposure intensity is lower or higher than the fiber material damage threshold [3]. However, the long-term instability under high temperature (up to 400 °C) has limited applications of type-I FBG in the high temperature field. 

In recent years, special FBG types such as type-II fiber grating, regenerated fiber grating (RFBG) and sapphire fiber grating (SFBG) have become hot research topics because of their performance at high temperature (higher than 400 °C) [4,5,6,7,8]. In 2012, the temperature and strain characterizations of seed and regenerated gratings with and without post-annealing was tested under the temperature of 1100 °C [9]. In 2015, sapphire fiber FBGs for high temperature applications were studied up to 1900 °C [10]. In 2016, the type-II gratings within silica suspended-core microstructure optical fibers fabricated by femtosecond lasers were tested at temperatures up to 1300 °C [11]. However, some of these FBGs still have certain limitations, such as the inscription difficulty of SFBGs, and the frangibility and low reflection intensity of RFBGs after repeated annealing. Therefore, researchers have focused on silica-based type-II fiber gratings in recent years.

In previous studies [5,8], theoretical research showed that the wavelength shift of type-II FBGs was mainly determined by the elastic coefficient, thermal expansion coefficient (CTE) and thermo-optical coefficient (TOC). However, the correlation coefficients of silica-based FBGs would no longer be constant at high temperatures, and the known sensing parameters were no longer viable in this situation. Meanwhile, calibration of FBG sensors at high temperatures before any measurements remains an unsolved problem. 

Therefore, how to measure the CTE and TOC of silica-based FBGs at high temperatures is an attractive research topic. The traditional measurement method for the TOC and CTE of transparent materials is the prism method [12]. Domenegueti et al. developed a single arm double interferometer for the simultaneous measurement of linear TOC and CTE of solid (silica, BK7, SF6) and liquid (water, ethanol and acetone) samples [13]. Li et al. used an approximately 8-layer graphene diaphragm to fabricate an optical fiber extrinsic FP interferometric sensor which could measure the CTE of a graphene diaphragm [14]. In previous work, we demonstrated a hybrid sensor fabricated by an etching method to measure the CTE and thermo-optical coefficient of silica-based type-I FBG in the temperature range of 30–273 K [15]. However, little research has been done on the temperature coefficient measurement of type-II silica-based FBGs at ultrahigh temperatures.

In this work, we successfully obtained the CTE and TOC of type-II FBGs through a FFPI-FBG hybrid sensor, which was fabricated by infrared (IR) femtosecond (fs) laser micromachining and pulsed radiation with a phase mask. The CTE and TOC of silica-based FBGs has been simultaneously calculated by measuring the wavelength of a type-II FBG and FFPI cavity length. The data accuracy has been carefully examined by comparing with the relevant references

## 2. Principle and Structure of the Sensor

**Figure 1:**
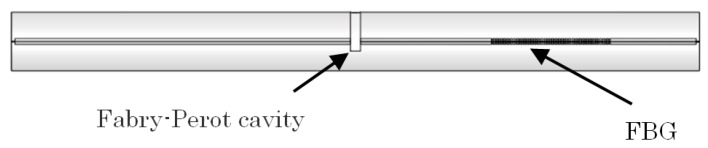
Schematic structure of the FFPI-FBG hybrid sensor.

Figure 1 shows the schematic structure of the hybrid sensor. It is composed of an inline FFPI cavity and a type-II FBG. 

**Figure 2:**
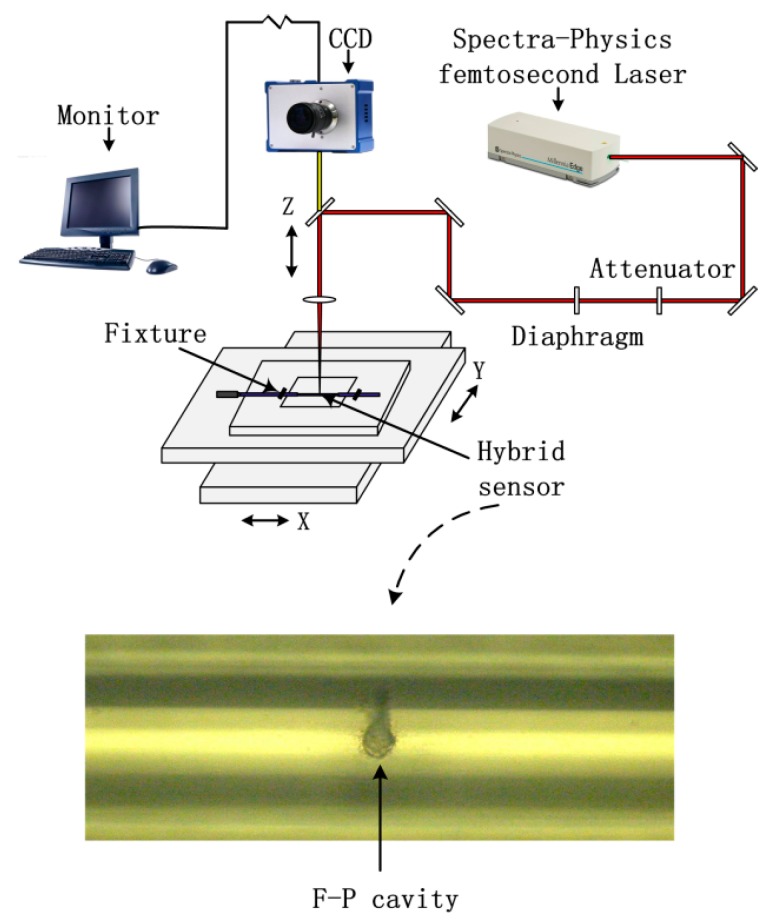
Schematic structure of the femtosecond laser 3D micromachining system and the digital microscope image of FP cavity fabricated.

**Figure 3:**
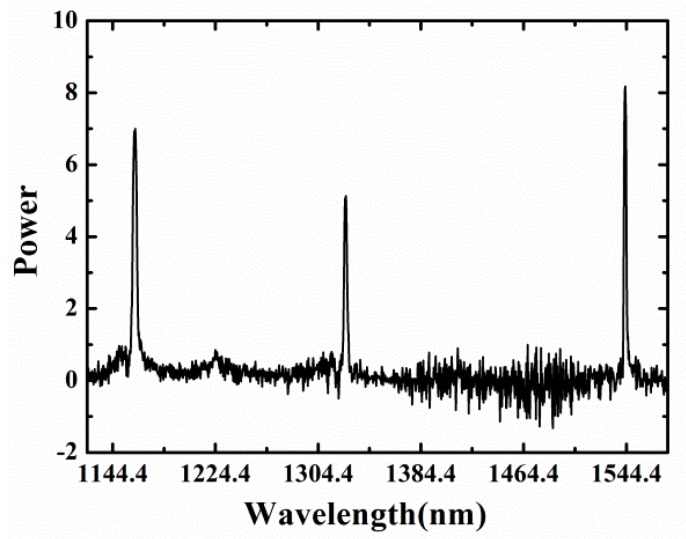
High-order spectra of the type-II Bragg grating structure.

As shown in Figure 2, the inline FFPI and type-II FBG were both fabricated by a fs laser 3D micromachining system which was composed of four parts, a fs laser (Spectra-Physics Inc., Santa Clara, CA, USA), external optical path, CCD monitoring system (SigamaKoki Inc., Tokyo, Japan) and 3D working platform (Newport Inc., Franklin, MA, USA). The single-mode fiber (SMF) was fused silica HIPOSH^®^ fiber (YOFC Ltd., Wuhan, China, with core/cladding of 9 µm/125 µm). The pulse width and repetition rate of the fs laser were 120 fs and 1 kHz, respectively. The 3D working platform had a movement range of ±100 ±100 ±25 mm (in the X,Y,Z directions, respectively) and the movement accuracy was 1 μm. The pulse energy was controlled by a tunable attenuator. The FBG inscription by the fs laser is a nonlinear and multiphotonic process for optical fibers [16]. The spectra of type-II FBGs was measured by using a supercontinuum source (YSL Photonics Inc., Wuhan, China) with an output wavelength range from 1100 nm to 1600 nm and an optical spectrum analyzer (OSA) (Yokogawa Inc., Tokyo, Japan) with resolution of 0.02 nm. Figure 3 shows that the type-II FBG had three high reflectivity resonances in the wavelength range from 1100 to 1600 nm. The FWHM of the FBG for all three orders (1.1 μm, 1.3 μm 1.5 μm) were 2.58 nm, 1.82 nm, and 0.84 nm, respectively. The pulses used for type-II FBG inscription were 3 mm radius, with 0.6 mJ pulse energy at 1000 Hz repetition rate. The average light power intensity in the focal line was about 10^13^ W/cm^2^. The pulses used for FFPI ablation were 3 mm radius, with 0.8 mJ pulse energy at 100 Hz repetition rate. The laser focusing mode has also been changed from line mode to point mode.

Through the cooperation of the 3D platform, the micro-machining of FFPI cavity was carried out around the type-II FBG. It could be seen from the digital microscope (VHX-100) that the interface of the FFPI cavity made by fs laser was coarse and irregular (Figure 2). Then a discharge arc was used to make the cavity interface clean and flat with a fusion splicer (Fujikura Inc., Tokyo, Japan).

**Figure 4:**
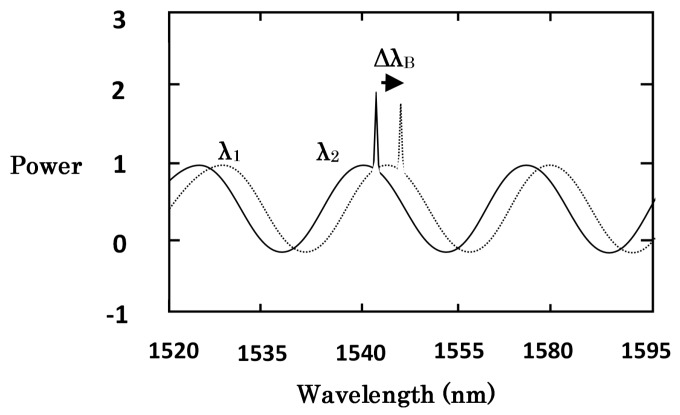
Transmission spectrum (1.5 µm) of the hybrid FFPI-FBG sensor.

Figure 4 showed the transmission spectrum (1.5 µm) of the hybrid sensor. The wavelength shifts of transmission spectra (1.5 µm) were recorded by a self-manufactured optical spectrum demodulation (OSD) system (Bayspec Inc., San Jose, CA, USA), in which the acquisition rate was 8 kHz and wavelength resolution was 0.1 pm. The spectral period change of FFPI was mainly determined by the micro-cavity length. The wavelength of the FBG and the cavity of the FFPI would change with the increase of environment temperature. 

The Bragg resonance shift $\Delta \lambda_{\left(B,m\right)}$ equation of the high order type-II FBG could be expressed as:

(1) $$\frac{\Delta \lambda_{\left(B,m\right)}}{\lambda_{\left(B,m\right)}}=(\xi +\alpha_{m})\times \Delta T$$

where $\lambda_{\left(B,m\right)}$ was the center wavelength of FBG, m was the diffracted order, ξ was the CTE, $\alpha_{m}$ was the TOC of the fiber grating at the wavelength $\lambda_{\left(B,m\right)}$, ΔT meant the variation of temperature.

Where $\lambda_{1}$ and $\lambda_{2}$ were the wavelength at the two adjacent peaks of the interference fringes [17]. The cavity length L of the FFPI could be calculated as:

(2) $$L=\frac{\lambda_{1}\lambda_{2}}{2(\lambda_{2}-\lambda_{1})}$$

The cavity variation ΔL could be obtained by the Equation (2) since the temperature change, so the CTE in different range could be expressed by:

(3) $$\xi =\frac{\Delta L}{L\Delta T}$$

The CTE measured by FFPI was the CTE of the fiber cladding since the fiber core was etched. The main raw material of SMF (YOFC HIPOSH^®^ fiber) was fused silica and the material composition and structure in SMF was SiO_2_-GeO_2_-F. Normally only GeO_2_ was doped in the central core and only F in the cladding. For PCVD optical fiber, F could be introduced in the core to reduce the water peak. The optical fiber was a cylindrical structure, with a 9 μm diameter core, surrounded by a cladding with 125 μm diameter. The dopant concentrations must be very tiny (nearly 0.1%) to get the perfect viscosity match between the core and cladding. Because the raw material was consistent and doping amount was very tiny, combined with good manufacturing process, the CTEs of the core and cladding could be regarded as approximately the same [18]. 

Thus the TOC could be expressed as:

(4) $$\alpha_{m}=\frac{\Delta \lambda_{\left(B,m\right)}}{\lambda_{\left(B,m\right)}\Delta T}-\xi .$$

The TOC of silica-based FBG was the effective thermo-optic coefficient of the modes, was different from the TOC of the fiber material.

## 3. Experiment

**Figure 5:**
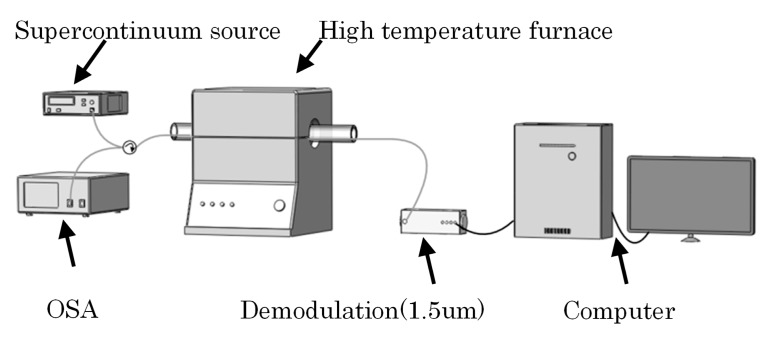
High temperature experimental set-up.

The schematic setup of the high temperature experiment was shown in Figure 5. Three FFPI-FBG hybrid sensors were placed close to the thermocouple in a tubular calibration furnace (OTF-1200X, KEJING Ltd., Changzhou, China). 

The tubular calibration furnace adopted PID controls with a temperature precision of 1 °C and had a maximum temperature of 1200 °C. The thermocouple near the hybrid sensors was used as a reference and for the standard temperature measurement. During the test, the furnace tube was in a vacuum state to avoid the influence of air.

The wavelength shift of resonance in 1.3 μm of type-II FBG was recorded by an OSA (Yokogawa Inc.). The supercontinuum source and OSA were connected to the two input ports of a 3 dB fiber coupler respectively. One end of the hybrid sensor in the furnace tube was connected to the output port of the coupler and the other end of the hybrid sensor was connected to a self-manufactured OSD (Bayspec Inc.). The temperature started from 30 to 50 °C, then rose to 1100 °C with a step of 50 °C and the speed of 10 °C/min. In each setting temperature point, the temperature was holding 20 min to ensure the heat exchange and eliminated the influence of the temperature fluctuation.

**Figure 6:**
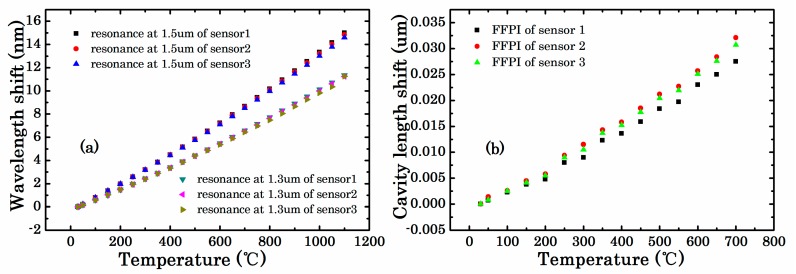
(**a**) Bragg wavelength shift and (**b**) FFPI cavity length change versus temperature at high temperatures.

The high temperature response of the type-II FBG and FFPI are plotted in Figure 6, respectively. The results showed that shift in Bragg wavelengths was nonlinear from 30 to 1100 °C, and the cavity length change was also nonlinear from 30 to 700 °C. The data at the temperature of 30 °C was defined as the initial values. The initial values of type-II FBGs were (1543.6734 nm, 1325.56 nm), (1543.3413 nm, 1325.272 nm), and (1543.8212 nm, 1325.691 nm), respectively. Also the initial cavity lengths of FFPI were 63.3994 μm, 71.3354 μm, and 69.1103 μm, respectively. The test temperature was holding 4 h at 1100 °C and the reflection spectra of the three type-II FBGs did not change throughout the testing process. The peak intensity of the three FFPI data was declined from nearly −53 dBm to −70 dBm when the temperature exceeds 700 °C. The OSD was unable to complete the peak search. The FFPI cavity data could hardly keep stable and fluctuated within a dozen microns. This phenomenon could be attributed to the uneven surface of the FP micro-cavity that led to an irregular expansion at such high temperatures, leading to larger intensity loss.

## 4. Results and Discussion

**Figure 7:**
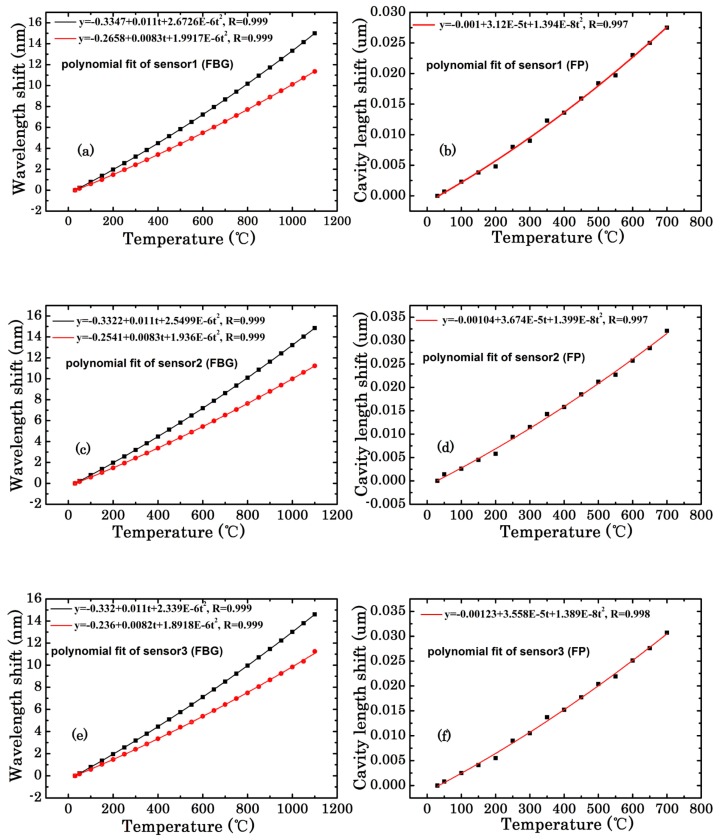
The quadratic polynomial fit curves of Bragg wavelength change versus temperature (**a**) FBG 1 (**c**) FBG 2 (**e**) FBG 3 and FFPI cavity length change versus temperature (**b**) FP1 (**d**) FP2 (**f**) FP3.

The dotted lines in Figure 7 are the quadratic polynomial fit curves of the Bragg wavelength and FFPI cavity length changes at different temperatures, respectively.

For the type-II FBG at the temperature ranging from 30 to 1100 °C, the polynomial expressions were as follows:

(5) $$\Delta \lambda_{\left(1.5um,1\right)}=-0.3347+0.011T+2.6726E-6T^{2},$$

(6) $$\Delta \lambda_{\left(1.5um,2\right)}=-0.3322+0.011T+2.5499E-6T^{2},$$

(7) $$\Delta \lambda_{\left(1.5um,3\right)}=-0.332+0.011T+2.339E-6T^{2},$$

(8) $$\Delta \lambda_{\left(1.3um,1\right)}=-0.2658+0.0083T+1.9917E-6T^{2},$$

(9) $$\Delta \lambda_{\left(1.3um,2\right)}=-0.2541+0.0083T+1.936E-6T^{2},$$

(10) $$\Delta \lambda_{\left(1.3um,3\right)}=-0.236+0.0082T+1.8918E-6T^{2}.$$

For the FFPI at the temperature ranging from 30 to 700 °C:

(11) $$\Delta L_{1}=-0.001+3.12E-5T+1.394E-8T^{2},$$

(12) $$\Delta L_{2}=-0.00104+3.674E-5T+1.399E-8T^{2},$$

(13) $$\Delta L_{3}=-0.00123+3.558E-5T+1.389E-8T^{2},$$

where *T* was in Celsius.

By differentiating Equation (3) and the average calculation of Equations (11)–(13), the CTE could be given by:

(14) $$\xi =\frac{\Delta L}{L\Delta T}=(0.0041T+5.07)E-7.$$

When the temperature was between 30 and 700 °C, the relationship between the CTE and temperature could be described as a linear curve. Differentiating Equation (1) and the average calculation of Equation (5) to Equation (10), the TOC and CTE could be deduced corresponding to the equation as follows:

(15) $${\left(\alpha +\xi \right)}_{1.5um}=\frac{\Delta \lambda_{B}}{\lambda_{B}\Delta T}=(0.00326T+7.12)E-6,$$

(16) $${\left(\alpha +\xi \right)}_{1.3um}=\frac{\Delta \lambda_{B}}{\lambda_{B}\Delta T}=(0.00292T+6.23)E-6.$$

Thus, the TOC could be acquired from Equation (4):

(17) $$\alpha_{1.5um}=(0.00285T+6.61)E-6,$$

(18) $$\alpha_{1.3um}=(0.00251T+5.723)E-6,$$

where *T* was in Celsius and temperature was between 30 °C and 700 °C. The relationship between the TOC and temperature also could be described by a linear curve. 

When the temperature was between 30 °C and 100 °C, the CTE was between 5.19 × 10^−7^/°C and 5.48 × 10^−7^/°C, TOC $\alpha_{1.5um}$ was between 6.69 × 10^−6^/°C and 6.89 × 10^−6^/°C, and $\alpha_{1.3um}$ was between 5.8 × 10^−6^/°C and 5.97 × 10^−6^/°C, which agreed well with the theoretical values at room temperature [19,20,21]. Thus, the curve equation could be used for silica-based FBG standardization procedures at high temperatures. Combined with metal coating technology, it strongly indicated that such a hybrid sensor also had great potential in characterizing the CTE of related metal at high temperatures. A metal coating (gold, copper) could be sputtered on the surface of bare SMF, using the ultrahigh vacuum magnetron sputter system. The thickness of the metal film would be larger than 10 μm and it would distribute homogeneously on the fiber surface. Then femtosecond laser processing could be employed to ablate the FFPI on the metal-coated fiber. The CTE of SiO_2_ was much less than that of metal, so one could calculate the CTE of the coated metal through the FFPI cavity changes.

## 5. Conclusions

The CTE and TOC of silica-based type-II FBGs have been tested by using a hybrid FFPI-FBG sensor fabricated by an IR-fs laser. The high temperature characteristics of silica-based type-II FBGs were tested from 30 to 1100 °C. By analyzing of the data, the formula for the CTE and TOC from 30 °C to 700 °C has been determined. The test values agreed well with the theoretical value at room temperature. Such a hybrid sensor made with IR-fs laser should be a highly promising platform for a variety of high temperature sensing applications.
